# Comment on “Surgically Resected Gall Bladder: Is Histopathology Needed for All?”

**DOI:** 10.1155/2016/8607814

**Published:** 2016-07-17

**Authors:** Savio George Barreto

**Affiliations:** Department of Gastrointestinal Surgery, Gastrointestinal Oncology, and Bariatric Surgery, Medanta Institute of Digestive and Hepatobiliary Sciences, Medanta-The Medicity, Gurgaon 122018, India

I read with interest the manuscript by Talreja and colleagues [1] that questions the need for routine histopathological examination of the “apparently normal” gall bladder following cholecystectomy based on their retrospective examination of their data in which 11 patients (with gall bladder cancer) out of 964 (patients who underwent a cholecystectomy during the study period) had either preoperative imaging or intraoperatively visible gross features of wall thickening. This study is not the first [2] and it will certainly not be the last to raise this contentious issue.

The problems with accepting the inferences of Talreja and colleagues are manifold. The first issue is that the authors themselves reported that only 55% of those with a cancer had suspicious thickening of the gall bladder on preoperative imaging. This means that 45% of patients with cancer were not detected on preoperative imaging. Secondly, only 6 patients (55%) with cancer had polypoidal lesions or ulcers in addition to thickening of the wall. This is in comparison to wall abnormalities being detected in 43% of the entire cohort!

We are aware that the incidence of gall bladder cancer is not uniform around the world with some regions demonstrating a higher incidence than others [3]. However, we all agreed that the outcome of gall bladder is uniformly dismal irrespective of race, religion, or geographical location [3].

There has been a conscious effort to try to understand the disease and how it develops [4–6]. However, all that we can state with certainty at the present time is that our best chance to cure or treat gall bladder cancer is to detect the disease early [7] when it is amenable to curative resection (lymphadenectomy and liver resection) with or without the need for adjuvant therapy [8]. We know that the survival following gall bladder cancer is inversely proportional to the extent of disease with even metastases to a solitary lymph node signalling poor outcomes [8].

Talreja and colleagues [1] put forth arguments against routine histopathological examination citing time invested by the pathologist and the financial implications of these “rather fruitless” pathological examinations. I have encountered patients presenting with vague upper abdominal symptoms a few months to a year after an apparently uneventful cholecystectomy in which the gall bladder was not submitted for pathological examination for reasons not dissimilar to those cited by Talreja and colleagues [1]. Ironically, the diagnosis of diffuse metastatic disease is reached after a battery of tests, including immunohistochemistry, conclusively implicating the erstwhile gall bladder.

Thus, I wish to assert that the cost of a pathological examination cannot be equated with the cost of a life lost, and the time spent by the pathologist in examining the gall bladder specimen cannot even come close to the time that is lost by the patients afflicted with gall bladder cancer and their loved ones.
